# Post‐extubation dysphagia and dysphonia amongst adults with COVID‐19 in the Republic of Ireland: A prospective multi‐site observational cohort study

**DOI:** 10.1111/coa.13832

**Published:** 2021-07-18

**Authors:** Julie Regan, Margaret Walshe, Sarah Lavan, Eanna Horan, Patricia Gillivan Murphy, Anne Healy, Caoimhe Langan, Karen Malherbe, Breda Flynn Murphy, Maria Cremin, Denise Hilton, Jenni Cavaliere, Alice Whyte

**Affiliations:** ^1^ Department of Clinical Speech and Language Studies Trinity College Dublin Dublin Ireland; ^2^ Speech and Language Therapy Department St. James’ Hospital Dublin Ireland; ^3^ Speech and Language Therapy Department Tallaght University Hospital Dublin Ireland; ^4^ Speech and Language Therapy Department Mater Misericordiae University Hospital Dublin Ireland; ^5^ Speech and Language Therapy Department Beaumont Hospital Dublin Ireland; ^6^ Speech and Language Therapy Department St. Vincent’s University Hospital Dublin Ireland; ^7^ Speech and Language Therapy Department Galway University Hospital Galway Ireland; ^8^ Speech and Language Therapy Department Midland Regional Hospital Tullamore & Portlaoise Offaly Ireland; ^9^ Speech and Language Therapy Department University Hospital Kerry Tralee Ireland; ^10^ Speech and Language Therapy Department Cavan General Hospital Cavan Ireland; ^11^ Speech and Language Therapy Department University Hospital Waterford Waterford Ireland; ^12^ Speech and Language Therapy Department Naas General Hospital Naas Ireland

**Keywords:** COVID‐19, dysphagia, dysphonia, intubation, post‐extubation, speech and language therapy, swallowing, voice

## Abstract

**Objectives:**

This study aims to (i) investigate post‐extubation dysphagia and dysphonia amongst adults intubated with SARS‐COV‐2 (COVID‐19) and referred to speech and language therapy (SLT) in acute hospitals across the Republic of Ireland (ROI) between March and June 2020; (ii) identify variables predictive of post‐extubation oral intake status and dysphonia and (iii) establish SLT rehabilitation needs and services provided to this cohort.

**Design:**

A multi‐site prospective observational cohort study.

**Participants:**

One hundred adults with confirmed COVID‐19 who were intubated across eleven acute hospital sites in ROI and who were referred to SLT services between March and June 2020 inclusive.

**Main Outcome Measures:**

Oral intake status, level of diet modification and perceptual voice quality.

**Results:**

Based on initial SLT assessment, 90% required altered oral intake and 59% required tube feeding with 36% not allowed oral intake. Age (OR 1.064; 95% CI 1.018–1.112), proning (OR 3.671; 95% CI 1.128–11.943) and pre‐existing respiratory disease (OR 5.863; 95% CI 1.521–11.599) were predictors of oral intake status post‐extubation. Two‐thirds (66%) presented with dysphonia post‐extubation. Intubation injury (OR 10.471; 95% CI 1.060–103.466) and pre‐existing respiratory disease (OR 24.196; 95% CI 1.609–363.78) were predictors of post‐extubation voice quality. Thirty‐seven per cent required dysphagia intervention post‐extubation, whereas 20% needed intervention for voice. Dysphagia and dysphonia persisted in 27% and 37% cases, respectively, at hospital discharge.

**Discussion:**

Post‐extubation dysphagia and dysphonia were prevalent amongst adults with COVID‐19 across the ROI. Predictors included iatrogenic factors and underlying respiratory disease. Prompt evaluation and intervention is needed to minimise complications and inform rehabilitation planning.

**Key Points**

1. Post‐extubation dysphagia and dysphonia are multifactorial and can lead to prolonged ICU stay, prolonged tube feeding, aspiration pneumonia and increased morbidity and mortality.
2. In this multi‐site prospective cohort study across eleven acute hospitals, 90% of adults required an altered oral diet post‐extubation and 36% were not allowed oral intake based on SLT evaluation. Sixty‐six per cent presented with post‐extubation dysphonia.
3. Age, proning and pre‐existing respiratory disease were predictors of post‐extubation oral intake status, whereas intubation injury and pre‐existing respiratory disease were predictors of post‐extubation dysphonia.
4. Over a third (37%) required dysphagia intervention post‐extubation, whereas 20% needed intervention for voice.
5. Dysphagia and dysphonia persisted in 27% and 37% cases, respectively, at hospital discharge, indicating that speech and language therapists should be included in outpatient multidisciplinary COVID clinics in the community.

## 1 BACKGROUND

The SARS‐CoV‐2 virus (termed COVID‐19) is a novel respiratory virus, which has led to an international pandemic. COVID‐19 has resulted in an unprecedented number of critically ill adults, which has overwhelmed intensive care unit (ICU) services worldwide. Endotracheal intubation and mechanical ventilation have been a central management procedure for critically ill patients with COVID‐19 in ICU settings.

Post‐extubation dysphagia (PED) and dysphonia are common in critical care patients,^1^, ^2^ and recent research has highlighted PED and dysphonia within the COVID‐19 population.^3^, ^4^ In a single‐centre observational cohort study, 79% of adults referred to SLT over a two‐month period during COVID‐19 had been intubated and all patients who had persistent dysphagia at discharge had been intubated.^3^ In another single‐centre study, 50% (*N* = 204) adults admitted into the intensive care unit with COVID‐19 infection were referred to SLT for a swallow assessment.^4^ Of these patients, 33% required diet modification and 67% were not allowed oral intake.^4^

Iatrogenic causes of dysphagia include prolonged intubation^5^ and intubation injury including laryngeal oedema, granulations, ulceration and vocal cord immobility.^6^ In a recent study of twenty patients with COVID‐19 infection who underwent laryngeal endoscopy, the most common laryngeal complications were voice‐related complaints, breathing and swallowing.^6^ All participants who underwent laryngoscopy in this study presented with abnormal findings, and the most common diagnoses were vocal cord immobility, posterior glottic stenosis and subglottic stenosis.^6^ The majority of these patients had been intubated with an average duration of three weeks during their inpatient admission. Moreover, all patients who had been proned during intubation presented with glottal pathology.^6^

Tracheostomy insertion can lead to aspiration risk and difficulties managing secretions.^7^ In a recent prospective study involving forty‐one adults with COVID‐19 infection who had a tracheostomy inserted post‐extubation, 19% had severe pathology on laryngeal examination.^7^ Of note, the vast majority of participants had the tracheostomy inserted beyond fifteen days of oral intubation.^7^ Over half of the participant group presented with dysphonia whereas 30% reported dysphagia, although 83% were on a normal diet.^7^

Other potential factors contributing to PED are delirium,^8^ proning,^6^, ^9^ disuse atrophy and critical illness neuropathy or myopathy during ICU stay ^10^ and neurological manifestations of COVID‐19.^11^ Central and peripheral nervous system complications of COVID‐19 include stroke, encephalitis and Guillain‐Barre syndrome. These can damage the neurological swallow network, contributing to dysphagia amongst COVID‐19 survivors.^11^ PED is associated with worse outcomes in ICU including aspiration pneumonia, prolonged tube feeding, delayed initiation of oral intake, prolonged hospitalisation and increased morbidity and mortality.^12^, ^13^

Dysphonia is another recognised complication of intubation reported amongst adults with COVID‐19.^3^ Recent research during the COVID‐19 pandemic highlighted that 56% of adults with dysphonia had persistent impairment at hospital discharge.^3^ Another study found voice difficulties were the most common laryngeal complication amongst adults with COVID‐19.^6^ Post‐extubation dysphonia results from vocal cord immobility, laryngopharyngeal reflux, granuloma, reduced breath support for phonation and vocal cord fatigue. Endotracheal tube (ETT) size^14^ and cuff pressure during intubation^15^ have been identified as risk factors for post‐extubation dysphonia. Age and duration of intubation has also been linked to prolonged dysphonia post‐extubation in previous critical care research.^16^ Trachesotomy can also cause laryngeal complications,^17^ and dysphonia has also been reported in over half of adults with COVID‐19 who had a tracheostomy inserted post‐extubation.^7^

The impact of intubation as part of COVID‐19 management on swallowing and voice is unclear internationally, as are the dysphagia and dysphonia intervention needs within this population.^18^ This study aims to characterise the presence and degree of post‐extubation voice and swallowing difficulties amongst adults requiring intubation as part of medical management of COVID‐19 in the Republic of Ireland (ROI) during the first wave of the pandemic. Specific research objectives are to (i) explore the presence, degree and trajectory of dysphonia and dysphagia post‐extubation in adults with COVID‐19 across the ROI between March and June 2020 inclusive; (ii) identify variables, which predict post‐extubation oral intake status and voice quality and (iii) determine SLT evaluation and intervention indicated and provided to this cohort.

## 2 METHODS

### 2.1 Study design

This multi‐site prospective observational cohort study is reported according to the STROBE guidelines for observational cohort studies.^19^ Ethical approval for this study was obtained from National Research Ethics Committee (NREC) (20‐NREC‐COV‐051).

### 2.2 Settings

In this multi‐centre observational cohort study, speech and language therapists from eleven acute hospitals across Ireland participated.

### 2.3 Participants

All adults admitted into a participating acute hospital in the ROI with COVID‐19 and referred to SLT were included. Inclusion criteria were (i) confirmed COVID‐19 positive based on polymerase chain reaction (PCR) test; (ii) intubated during inpatient stay as part of COVID‐19 treatment; (iii) referred to SLT during hospital admission and (iv) consent obtained. Exclusion criteria were (i) age 16 years or younger; (ii) unconfirmed COVID‐19 infection; (iii) no consent and (iv) intubation for reasons other than COVID‐19 infection.

### 2.4 Independent variables

Demographic data included age, gender, pre‐admission medical comorbidities and premorbid swallow status. During hospital admission, data on neurological manifestations of COVID‐19 were captured by speech and language therapists from hospital records. The patient's most recent chest X‐ray at time of initial SLT assessment was rated by speech and language therapists based on medical entries into clinical notes using a validated five‐point ordinal scoring system provided in dataset dictionary.^20^

Intubation variables collated from healthcare records were grade of intubation based on the Cormack and Lehane system,^21^ number of endotracheal tubes (ETT) used, maximum ETT cuff pressure during intubation, number of failed extubations and presence of intubation injury as reported in clinical notes. Data on proning and proning‐related injury were collected. Tracheostomy insertion, tracheostomy type and size, and time to decannulation were also included. Length of ICU stay (LOSICU) and hospital length of stay (LOS) were recorded.

### 2.5 Swallow and voice outcomes

Swallowing and voice outcomes were influenced by curtailed access during the COVID‐19 pandemic to instrumental assessments typically used in intensive care settings such as fibreoptic endoscopic evaluation of swallowing (FEES). Presence and severity of PED was measured by speech and language therapists using the Functional Oral Intake Scale (FOIS).^22^ The FOIS is a validated 7‐point ordinal rating scale with high inter‐rater reliability.^22^ A second proxy dysphagia measure used by speech and language therapists was food and fluid consistencies required for dysphagia management, using the International Dysphagia Diet Standardisation Initiative (IDDSI).^23^ Voice quality was evaluated by speech and language therapists using the overall Grade (G) score from the GRBAS scale.^24^ The scale has established high rater reliability and is widely used in clinical research.^25^

### 2.6 Data sources/management

One nominated speech and language therapist from each hospital site was responsible for data entry at each location. A dataset and dataset dictionary were emailed to named speech and language therapists from each participating site. Speech and language therapists were instructed to populate the dataset prospectively and return the anonymised data to the first authors for analysis.

### 2.7 Bias

To minimise observer bias, all clinicians used outcome measures routinely used in clinical practice with established rater reliability. Clear rules and procedures were in place for data collection, and data were clearly defined in a data dictionary provided to all settings. Merged data were anonymised to researchers.

### 2.8 Study size

Patients who meet the eligibility criteria over the three‐month data collection period were included in the study. The study size was determined by the prevalence of cases and in particular those needing respiratory support. Statistical advice was obtained regarding recruitment numbers and statistical power for the sample.

### 2.9 Statistical analysis

Descriptive statistics were reported using medians and interquartile range (IQR) for continuous data. Categorical variables were presented as frequency (percentage). Variables were tested for normality using the Shapiro‐Wilk test. To establish associations between dependent and independent variables, Spearman's rho correlations were conducted. To determine the trajectory of dysphagia and dysphonia from initial SLT assessment to SLT discharge, medians of ordinal dependent variables at both time points were compared using two‐tailed Wilcoxon signed rank tests.

To determine independent predictors of oral intake status at time of initial SLT assessment, a binary logistic regression was used. The seven‐point ordinal FOIS rating scale was divided into feeding tube reliant (FOIS Levels 1–3) and not tube feeding reliant (FOIS Level 4–7) categories as the binary dependent variable. To prevent over‐fitting the model, six independent variables were selected for oral intake status (age, duration of intubation, proning, neurological manifestations, maximum cuff pressure and history of respiratory disease).

For voice quality, an ordinal logistic regression was completed with the overall (G) four‐point ordinal GRBAS rating as the dependent variable. Six independent variables were selected for the voice regression model (intubation injury, proning, maximum cuff pressure, duration of intubation, number of comorbidities and history of respiratory disease).

For both regression models, independent variables were selected based on evidence from previous research and a visual review of the data. Where a significant association was identified between independent variables (eg duration of intubation and presence of tracheostomy), only one was selected for a model. Mean imputation was made for one independent variable (maximum cuff pressure) as it was missing 24/100 cases. Model fits were confirmed using likelihood ratio chi‐squared tests. A two‐sided α of less than 0.05 was considered statistically significant. Statistical analyses were completed using the SPSS (v26) software.

## 3 RESULTS

### 3.1 Participants

Data from 100 adults with PCR test confirmed COVID‐19 infection who were intubated across eleven acute hospitals in ROI and referred to SLT between March and June 2020 inclusive were included in analysis. The 100 adults (69% male) had a mean age of 62 years (age range 17–88 years). Further demographic details are in Table 1.

**TABLE 1:** Demographic data

Age	Mean	62 years
	Range	17–88 years
Gender	Males	69 males
	Females	31 females
Source of admission	Home	81%
	Residential setting	2%
	Transfer from other hospital	14%
	Transfer from rehab setting	1%
	Unknown	2%
Co‐morbidities	None	6%
	Minimum one comorbidity:	94%
	One comorbidity	34%
	Two	28%
	Three	20%
	Four	9%
	Five	3%
	Mean/median no of comorbidities:	2
	Medical comorbidities:	
	Respiratory disease (COPD/other respiratory disease)	21%
	Cardiology	34%
	Stroke	0%
	Progressive neurological	1%
	Dementia	0%
	Mental health condition	10%
	Head and neck cancer	0
	Cancer outside of head and neck	10%
	Diabetes	22%
	Obesity	29%
	Intellectual disability	1%
	Other	46%
Pre‐admission swallow status	Normal diet (FOIS Level 7)	96%
	FOIS Level 5	3%
	FOIS Level 1	1%
COVID−19 Neurological manifestations	Total:	37% (34/96)
	Stroke *n* = 2	
	Seizures *n* = 1	
	Impaired consciousness *n* = 20	
	Delirium *n* = 8	
Most recent	Abnormal	99%
Chest X‐ray at time of SLT initial assessment	Chest X‐ray classification (Taylor et al, 2015):	
	Patchy atelectasis and/bronchial wall thickening	8%
	Focal consolidation	8%
	Multifocal consolidation	74%
	Diffuse alveolar changes	2%
	Unknown	1%

Median duration of intubation was 14 days (IQR 8–19.5). Data on grade of intubation, number of ET tubes, maximum cuff pressure (76/100), number of failed extubations, proning and tracheostomy are captured in Table 2. Missing data for some of these variables were due to limited access to data across clinical settings.

**TABLE 2:** Ventilation data

Intubation details (where data is missing it is because data could not be obtained from local healthcare records)	Intubation	
	Median duration of intubation	14 days (IQR 8–19.5) (range 1–49)
	Median grade of intubation (*n* = 48)	1 (IQR) (range 1–2)
	Median no. of endotracheal tubes (*n* = 87)	1 (IQR 1–2)
	Median max cuff pressure (*n* = 76)	30 mmHg (IQR 30–35) (range 22–60)
	Median no failed extubations (*n* = 92)	0 (range 0–2)
	Intubation injury	22/100
	Oedema	11/22
	Stridor	3/22
	Vocal cord immobility	1/22
	Other	7/22
	Proning	
	Proning completed	61% (61/100)
	Median Proning duration (days)	4 days (IQR 2–11 days)
	Proning related injury	26% (16/61)
	Tongue lip or facial swelling	*n* = 9
	Pressure sores	*n* = 4
	Lip laceration	*n* = 1
	Herpes rash around mouth	*n* = 1
	Ulcer on chin/around mouth	*n* = 1
	Tracheostomy	
	Tracheostomy insertion (*n* = 100)	36%
	Percutaneous	27
	Surgical	9
	Median Tracheostomy size	8 (range 6–9)
	Mean time to decannulation (*n* = 34; 2 pts still with trach excluded)	24 days (SD: 16.11; range 3–71 days)
	Length of stay	
	Median Length of ICU Stay	20 days (IQR 11–34)
Median Length of Hospital Stay	38 days (IQR 28–68)

### 3.2 Presence, severity and trajectory of swallowing and voice outcomes post‐extubation

Median time between extubation and initial SLT evaluation was 4 days (IQR 2–11 days). Ninety per cent (*n* = 90) of patients presented with dysphagia (FOIS Level 1–6) at initial SLT assessment with 36% not allowed oral intake (FOIS Level 1) (Table 3). Median FOIS score at initial SLT assessment was 2.5 (SD 2.139; range 1–7) (*n* = 100). IDDSI fluid and food consistency findings are detailed in Table 3. A significant negative correlation was observed between oral intake status at initial SLT assessment as rated by the FOIS and ICU length of stay (ICULOS) (*r* = −.227; *p* = .028) and also between oral intake status and hospital LOS, indicating that the lower the FOIS score, the longer the LOS (*r* = −.363; *p* = .000).

**TABLE 3:** Trajectory of swallow and voice outcomes from initial assessment to SLT discharge (median timeframe 36.4 days; IQR 26–53 days)

Oral intake status (Functional oral intake scale)	Initial SLT assessment (*n* = 100)	Discharge (*n* = 95)
1: Tube dependent. Nothing by mouth.	36	4
2: Tube dependent with minimal attempts at food or liquid.	14	0
3: Tube dependent with consistent oral intake of food or liquid.	9	0
4: Total oral diet of a single consistency	2	0
5: Total oral diet with multiple consistencies, but requiring special preparation or compensations.	25	10
6: Total oral diet with multiple consistencies without special preparation, but with specific food limitations.	4	8
7: Total oral diet with no restrictions	10	73
Median (IQR)	2.5 (1–5)	7 (7–7)
Z score (*p* value)	−7.322 (*p* = .000)	

Fluid consistency (IDDSI fluid consistencies)	Initial SLT assessment (*n* = 100)	Discharge (*n* = 91)
0: Thin fluid	43	85
1: Slightly thick fluid	12	5
2: Mildly thick fluid	11	1
3: Moderately thick fluid	1	‐
4: Extremely thick fluid	3	‐
5: No fluids	30	‐
Median (IQR)	1 (0–5)	0 (0–0)
Z score (*p* value)	−6.023 (*p* = .000)	

Food consistency (IDDSI food consistencies)	Initial SLT assessment (*n* = 100)	Discharge (*n* = 91)
4: Puree	21	2
5: Minced and moist	15	1
6: Soft & bite sized	12	9
7: Regular	12	78
0: (none)	40	1
Median (IQR)	4 (0–5)	7 (7–7)
Z score (*p* value)	−8.976 (*p* = .000)	

Voice quality (GRBAS‐ G)	Initial SLT assessment (*n* = 99)	Discharge (*n* = 83)
0: No abnormality detected	34	63
1: Mild	31	12
2: Moderate	20	7
3: Severe	14	1
Median (IQR)	1 (0–2)	0 (0–0)
Z score (*p* value)	−5.619 (*p* = .000)	

Two‐thirds (66%; *n* = 99) of participants presented with post‐extubation dysphonia (GRBAS 1/+) with 14% in severe (rating 3) category (Table 3). A weak positive association was detected between GRBAS rating and LOS (*r* = .235; *p* = 022), indicating that the higher the GRBAS score (poor vocal quality), the longer the hospital LOS. Voice quality was not associated with ICULOS (*r* = .084; *p* = .428).

Oral intake status (median FOIS score) altered significantly from initial SLT assessment (FOIS score 2.5) to time of SLT discharge (median FOIS score 7) (*z* = −7.322; *p* = .000). A significant change was also observed in IDDSI fluid (*z* = −6.023; *p* = .000) and food (*z* = −7.52; *p* = .000) consistencies within the participant group from initial assessment to discharge (Table 3).

Median voice quality rating also altered significantly from initial SLT assessment (GRBAS score 1) to SLT discharge (GRBAS score 0) (*z* = −5.619; *p* = .000). Details regarding alteration in swallow and voice outcomes are in Table 3.

### 3.3 Variables predicting dysphagia and dysphonia at initial SLT assessment

In a multivariate model, statistically significant predictors of post‐extubation oral intake status included age (OR 1.064; 95% CI 1.018–1.112; *p* = .006), proning (OR 3.671; 95% CI 1.128–11.943; *p* = .031) and history of respiratory disease (OR 5.863; 95% CI 1.521–22.599; *p* = .010, Table 4).

**TABLE 4:** Independent variables predictive of post‐extubation oral intake status

Independent variable	B	SE	Odds ratio	95% CI for OR		*p* value
				Lower	Upper	
Age	.062	.0225	1.064	1.018	1.112	.006^*^
Maximum cuff pressure	−.001	.0448	.999	.915	1.091	.988
Proning	1.300	.6019	3.671	1.128	11.943	.031^*^
Neurological manifestations	1.049	.5804	.2856	.915	8.907	.071
History of respiratory disease	1.769	.6884	5.863	1.521	11.599	.01^*^
Duration of intubation	−.860	.5381	.423	.147	1.215	.110

^*^ Statistically significant at .05 level.

In a multivariate model, statistically significant predictors of post‐extubation voice quality were intubation injury (OR 10.471; CI 1.060–103.466; *p* = .044) and history of respiratory disease (OR 24.196; 95% CI 1.609–363.78; *p* = .021, Table 5).

**TABLE 5:** Independent variables predictive of post‐extubation voice quality

Independent variable	B	SE	Odds ratio	95% CI for OR		*p* value
				Lower	Upper	
Intubation injury	2.349	1.1687	10.471	1.060	103.466	.044^*^
Number of comorbidities	−.310	.5136	.733	.268	2.007	.546
Proning	1.328	.9441	3.775	.593	24.015	.159
Maximum cuff pressure	−.087	.0697	.916	.799	1.050	.209
Duration of intubation	−.113	.0668	.893	.784	1.018	.091
History of respiratory disease	3.186	1.3829	24.196	1.609	363.78	.021^*^

^*^ Statistically significant at .05 level.

### 3.4 SLT intervention needs and services provided

Over a third (*n* = 37/100) of patients required dysphagia intervention post‐extubation (Table 6). In 70% (26/37) of these cases, dysphagia intervention was implemented, although 19% (7/37) had it provided in adapted form due to infection risk related to the pandemic. In 11% (4/37) of cases, dysphagia intervention was indicated but could not be provided at point of service delivery due to the pandemic service constraints. A fifth (20%; *n* = 20/100) of participants required voice intervention post‐extubation. Within this subgroup, 55% (11/20) received standard voice intervention and 15% (3/20) received it in adapted form (Table 6). In 30% (6/20) cases, voice intervention was indicated but could not be implemented also due to pandemic service constraints.

**TABLE 6:** SLT intervention

	Dysphagia intervention	Number of participants implemented	Voice Intervention	Number of participants implemented
1	Oro‐facial exercises	3	Vocal cord adduction exercises	4
2	Sensory stimulation	2	Vocal function exercises	5
3	Masako manoeuvre	10	Vocal hygiene	9
4	Effortful swallow	12	Respiratory support for phonation	9
5	Supraglottic swallow	0	EMST	1
6	Pitch glide	3	Other	2
7	Mendelsohn manoeuvre	5		
8	Postural strategy	6		
9	Chin tuck against resistance	1		
10	EMST	1		
11	NMES	0		
12	Other	17		

Abbreviations: EMST, Expiratory muscle strength training; NMES, neuromuscular electrical stimulation.

## 4 DISCUSSION

In this study, 90% of patients intubated as part of COVID‐19 management across the ROI who were referred to SLT presented with new onset PED based on oral intake status. Over half (59%) required tube feeding based on SLT assessment, and over a third were not allowed oral intake post‐extubation. This high rate of PED compares with recent research.^3^, ^4^ Post‐extubation oral intake status was associated with length of ICU stay and hospital stay duration in this study, with reduced oral intake associated with longer duration of ICU and hospital admissions.

There was a threefold increase in impact on oral intake status with proning in this study. Lower cranial nerve paralysis and oropharyngeal oedema have previously been linked to proning, and cranial nerves IX to XII are hypothesised to be affected by proning.^6^, ^26^ Pre‐existing respiratory disease was also identified as a positive predictor of PED in this study. Adults with respiratory disease may already have altered respiratory swallow coordination, which could be exacerbated post‐extubation. There was approximately a 6% increase in the relative odds of oral intake status change per year of age in this study. Older people may have a pre‐existing presbyphagia, which pre‐disposes them to PED. Furthermore, frailty and sarcopaenia may also be prevalent amongst older people, which could contribute towards PED. In contrast to previous research,^5^ duration of intubation was not predictive of oral intake status in this study. This may be due to the fact that patients with tracheostomy were not excluded in this study, as researchers aimed to capture all adults with COVID‐19 post‐extubation. Additionally, prolonged intubation duration with COVID‐19 may explain contrasting findings to previous PED research.

There was a tenfold increase in impact on voice quality for those with intubation injury, which aligns with previous research.^2^, ^16^ This highlights the importance of post‐extubation endoscopy to evaluate vocal cord function in the ICU setting. Those with a history of respiratory disease were at threefold risk of impact on voice quality. Dysphonia is prevalent in adults with COPD which is due, in part, to altered pulmonary function.^27^ This alteration may be exacerbated post‐extubation, which may negatively impact on voice. In contrast to previous research,^15^ endotracheal cuff pressure was not associated with post‐extubation dysphonia in this study.

The number of adults receiving SLT intervention during hospital stay appeared low in this study, and some adults did not receive dysphagia and dysphonia intervention when indicated. This aligns with recent research,^4^ and it may relate to concerns regarding aerosol generated procedures during intervention as well as lack of instrumental evaluations which may have influenced the amount and type of intervention being offered during the first wave of the pandemic.^16^ Other influencing factors may be access to personal protective equipment, SLT services in ICU settings across ROI and local dysphagia training.

The rates of persistent dysphagia and dysphonia at hospital discharge mirror previous COVID‐19 research.^3^, ^4^ These subgroups may require long‐term rehabilitation due to ICU acquired muscle weakness, post‐intensive care syndrome (PICU) and neurological deficits. These figures are clear evidence that speech and language therapists should be core members of outpatient multidisciplinary COVID‐19 clinics.

Limitations to this study included missing data on oral health, delirium, grade of intubation and endotracheal tube size. Missing data were particularly difficult to access from intensive care records due to transmission risk. Patient‐reported outcomes would have been beneficial but not feasible post‐extubation given how medically unwell this cohort were. Validated scales to measure frailty and sarcopenia amongst adults intubated may have been useful, but again this was not feasible in the context of this study.

FEES was not available in ICU settings during the first wave of the pandemic due to international guidelines regarding transmission risk.^18^ FEES provides physiological data on secretions, pharyngeal sensation and aspiration or residue. While the validated outcomes used in this study are frequently employed in dysphagia research, they are not direct measures of swallow function. They instead capture the speech and language therapist's confidence in swallow function based on observed factors at the bedside including the acute status of the patient. It could be argued that oral intake is a more meaningful outcome compared to physiological swallow measures from the patient perspective. Nevertheless, endoscopic assessment of swallowing and intubation injury would be valuable to directly evaluate swallowing and to accurately evaluate the presence and nature of laryngeal injuries.^6^

Post‐extubation dysphonia and dysphagia research is needed from future pandemic waves to establish the impact of evolving intensive care management and mutating virus variants on voice and swallowing outcomes. Post‐discharge time points to capture longer term voice and swallowing difficulties would guide multidisciplinary service delivery in the community.

## 5 CONCLUSIONS

This study highlights the prevalence of post‐extubation dysphagia and dysphonia amongst adults intubated with COVID‐19. Awareness of the predictors of altered swallowing and voice quality post‐extubation will promote early in‐depth evaluation and monitoring during hospital stay. Prompt dysphagia and dysphonia evaluation and management is needed to minimise clinical and quality of life complications.

## DISCLOSURE STATEMENT

Authors have no disclosures to report.

## CONFLICTS OF INTEREST

None.

## AUTHOR CONTRIBUTION

J. Regan and M. Walshe designed the study, applied for ethical approval, analysed the data and wrote the paper. All other authors contributed to the study design, acquired and transferred data for analysis and contributed to data analysis and interpretation. All authors gave approval for paper to be submitted for publication.

## ETHICS STATEMENT

Ethical approval for this study was obtained from National Research Ethics Committee (NREC) (20‐NREC‐COV‐051).

## Data Availability

Authors are unable to share data due to ethical approval restrictions.
